# Combined primary carnitine deficiency with neonatal intrahepatic cholestasis caused by citrin deficiency in a Chinese newborn

**DOI:** 10.1186/s12887-020-02372-7

**Published:** 2020-10-13

**Authors:** Yiming Lin, Weihua Lin, Yanru Chen, Chunmei Lin, Zhenzhu Zheng, Jianlong Zhuang, Qingliu Fu

**Affiliations:** 1 Neonatal Disease Screening Center, Quanzhou Maternity and Children’s Hospital, 700 Fengze Street, Quanzhou, 362000 Fujian Province China; 2 Prenatal Diagnosis Center, Quanzhou Maternity and Children’s Hospital, 700 Fengze Street, Quanzhou, 362000 Fujian Province China

**Keywords:** Primary carnitine deficiency, Neonatal intrahepatic cholestasis caused by citrin deficiency, Newborn screening, Intrahepatic cholestasis, Ventricular septal defec

## Abstract

**Background:**

Primary carnitine deficiency (PCD) is an autosomal recessive disorder affecting the carnitine cycle and resulting in defective fatty acid oxidation. Neonatal intrahepatic cholestasis caused by citrin deficiency (NICCD) is an autosomal recessive disorder and one of the main causes of inherited neonatal cholestasis. Both PCD and NICCD are included in the current expanded newborn screening (NBS) targets.

**Case presentation:**

Targeted exome sequencing was performed on a Chinese proband, and Sanger sequencing was utilised to validate the detected mutations. The patient who was initially suspected to have PCD based on the NBS results presented with neonatal intrahepatic cholestasis and ventricular septal defect. Further investigations not only confirmed PCD but also revealed the presence of NICCD. Four distinct mutations were detected, including c.51C > G (p.F17L) and c.760C > T (p.R254X) in *SLC22A5* as well as c.615 + 5G > A and IVS16ins3kb in *SLC25A13*.

**Conclusions:**

This is the first reported case of PCD and NICCD occurring in the same patient. The dual disorders in a newborn broaden our understanding of inherited metabolic diseases. Thus, this study highlighted the importance of further genetic testing in patients presenting with unusual metabolic screening findings.

## Background

Primary carnitine deficiency (PCD, OMIM #212140) is an autosomal recessive disorder of fatty acid oxidation caused by mutations in the *SLC22A5* gene [1]. PCD is characterized by an estimated prevalence of 1:40,000–1:120,000 [2], with an extremely high frequency of 1:300 in the Faroe Islands [3]. Patients with PCD can suffer from skeletal or cardiac myopathy, muscle weakness, and hepatic encephalopathy [2]. Moreover, PCD patients have a lifetime risk of sudden death if left untreated [4]. PCD can be identified during newborn screening (NBS) by measuring free carnitine (C0) levels in dried blood spots [5]. Early diagnosis and treatment can prevent metabolic decompensation and possible death.

Neonatal intrahepatic cholestasis caused by citrin deficiency (NICCD, #OMIM 605814) is an autosomal recessive disorder caused by biallelic *SLC25A13* mutations [6]. NICCD is a pan-ethnic disorder with a high prevalence in East Asian populations. The incidence of NICCD in Japan is estimated on the basis of a carrier ratio (1:65) and is equal to 1:17,000 [7]. Our previous study, combined with genetic screening, revealed that the frequency of NICCD in five Chinese NBS programs was 1:26403 [8]. Patients with NICCD are characterised by neonatal intrahepatic cholestasis, hyperbilirubinemia, hepatomegaly, and variable liver dysfunctions, including fatty liver disease [9]. With timely treatment, the onset of NICCD usually resolves spontaneously before 1 year of age. However, few patients may present with severe hyperammonemia, hepatic encephalopathy, and liver failure, even requiring liver transplantation [10]. The elevated citrulline levels characteristic of NICCD can be detected by tandem mass spectrometry (MS/MS) during NBS. However, currently, such screening is not optimal due to the occurrence of false negatives [8, 11].

Here, we report a newborn who was initially suspected to have PCD based on the NBS results. Further investigations not only confirmed that the patient had PCD but also revealed the presence of NICCD. As a result, the biochemical, genetic, and clinical features of dual-inherited metabolic diseases were described in this patient.

## Case presentation

### Case report

This study was approved by the Ethics Committee of Quanzhou Maternity and Children’s Hospital. Written informed consent was obtained from the parents of the patient regarding the participation in the study as well as the use of obtained medical data for scientific research and publication. The proband was born by normal delivery at a gestational age of 41 weeks and 1 day, and her weight at birth was 3450 g. She was the first child of non-consanguineous parents originating from China. There was no significant family history of inherited metabolic diseases. NBS was performed on dried blood spots of the proband on day 5 of life via ACQUITY TQD MS/MS (Waters, Milford, MA, USA).

The initial NBS results showed that the patient had an extremely low C0 level equal to 3.49 μmol/L. The subsequent auxiliary biochemical tests confirmed the very low C0 level; however, they additionally revealed an abnormally high concentration of citrulline. Elevation of multiple amino acid levels, including methionine, arginine, and phenylalanine, was also observed. Moreover, serum total bile acid, total bilirubin, and direct bilirubin levels were significantly elevated, indicating the existence of cholestasis. Serum α-fetoprotein level was far beyond the reference range (Table 1) and the cardiac ultrasound revealed a ventricular septal defect (VSD) in the newborn.

**Table 1:** The biochemical, genetic, and clinical features of patient with PCD and NICCD

Patient’s information	5 d (NBS)	18 d	26 d	45 d	79 d	Reference range
C0 (μmol/L)	3.5	3.5	54.5	14.6	36.4	8.5–50
Citrulline (μmol/L)	28.8	277.2	565.6	73	51.5	6.0–34
Citrulline/Arg	3.2	4.3	5.3	1.5	1.5	0.3–6.5
Citrulline/Phe	0.7	2.5	5.7	1.2	1	0.05–0.7
Methionine (μmol/L)	28.2	69	231	66.4	42	8–38
Arginine (μmol/L)	9	64.1	106.9	49.1	35.3	1–50
Phenylalanine (μmol/L)	43.7	111.6	99.3	59.3	50.7	20–100
Total bile acid (μmol/L)			182.6	18.9	35.9	0–10
Total bilirubin (μmol/L)			339.8	226.8	37.7	5.1–19
Direct bilirubin (μmol/L)			31.8	18.2	15.9	0–6.8
ALT (U/L)			33	16	34	0–40
AST (U/L)			84	27	47	0–40
γ-GT (IU/L)			151	128	216	0–50
Ammonia (μmol/L)			47			10–47
α-Fetoprotein (ng/mL)			60,786.7	82,407.8	23,101.1	0–8.1
Total protien (g/L)			41.5	44.4	47.5	60–80
Hemoglobin (g/L)				95		110–116
Blood sugar (mmol/L)			4.2	4.6	5	3.8–6.1
CK (IU/L)			63			26–174
CK-MB (U/L)			15			0–35
Genotype	*SLC22A5*: c.51C > G + c.760C > T, *SLC25A13*: c.615 + 5G > A + IVS16ins3kb					
Clinical presentations	Intrahepatic cholestasis, ventricular septal defect					

*NBS* newborn screening, *d* day, *C0* free carnitine, *ALT* alanine transaminase, *AST* aspartate transaminase, *γ-GT* gamma-glutamyl transpeptidase, *CK* creatine phosphokinase, *CK-MB* creatine kinase isoenzyme

### Genetic analysis and targeted NGS

**Fig. 1:**
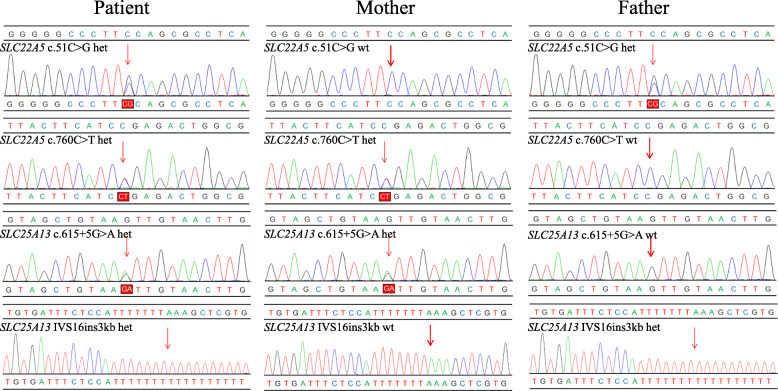
Pedigree verification of *SLC25A13* mutations by Sanger sequencing (the identified mutations are indicated with a red arrow)

Genetic testing was performed by Hangzhou Genuine Clinical Laboratory Co. Ltd. (Zhejiang, China). Genomic DNA was extracted from whole blood of the proband and her parents using a Qiagen DNA Blood Mini Kit (Qiagen®, Hilden, Germany). The DNA was subjected to NGS targeting a gene panel of 94 genes known to be associated with inherited metabolic diseases. The list of tested genes is included in the Supplementary material (Table S1). Then, Sanger sequencing was carried out to establish carrier status and confirm the detected alleles. Genetic testing showed the presence of four distinct mutations, including c.51C > G (p.F17L) and c.760C > T (p.R254X) in *SLC22A5* as well as c.615 + 5G > A and IVS16ins3kb in *SLC25A13*. All four mutations have previously been described as pathogenic in patients with PCD or NICCD [12, 13]. Sanger sequencing analysis confirmed that c.51C > G (p.F17L) and IVS16ins3kb were inherited from the father of the patient, while c.760C > T (p.R254X) and c.615 + 5G > A were inherited from her mother (Fig. 1). As a result, the patient was diagnosed with PCD combined with NICCD. Following the diagnosis, L-carnitine supplementation (150–300 mg/kg/day) was initiated, and breastfeeding was switched to a galactose-free and medium-chain triglyceride (MCT)-enriched formula. The biochemical and metabolic indicators gradually returned to normal levels after treatment.

## Discussion and conclusions

This study examined a newborn with an extremely low C0 level at NBS indicating PCD. It is interesting that the subsequent tests not only confirmed the reduced level of C0 but also revealed the elevated levels of multiple amino acids, particularly citrulline. Further biochemical tests showed abnormal liver function and cholestasis, indicating the presence of a second inherited metabolic disease. Molecular genetic analysis confirmed that the patient was affected by both PCD and NICCD. Thus, this study highlighted the importance of further genetic testing in patients presenting with unusual metabolic screening findings.

The persistently low C0 levels accompanied by the reduction of multiple acylcarnitine levels in the newborn, increased significantly after L-carnitine supplementation. Notably, the amino acid profile was normal at the time of NBS, and markedly elevated citrulline levels were not observed until the recall phase. If the additional testing was not performed due to suspicion of PCD, the patient with NICCD would have been missed in routine NBS. As our previous study revealed, more than half of the NICCD patients were missed in the MS/MS-based NBS program [8]. Therefore, incorporating genetic screening into the current NBS program can greatly improve the diagnosis of NICCD. A previous study has shown that high tyrosine levels in NICCD patients are associated with poor prognosis [14], whereas the tyrosine levels of our patient were consistently normal. All detected mutations in *SLC22A5* and *SLC25A13* are known to occur at high frequency in the Chinese population. The c.760C > T (p.R254X) in *SLC22A5* was previously reported as a founder mutation in the southern Chinese population [15], and IVS16ins3kb in *SLC25A13* is the second most common mutation in China [16].

Although both PCD and NICCD have a relatively high incidence in the Chinese population [17], these disorders rarely coexist in the same individual. To our knowledge, this is the first reported case of PCD and NICCD occurring in the same patient. Popek et al. reported a newborn diagnosed with glutaric aciduria type I combined with isobutyryl-CoA dehydrogenase deficiency; however, the latter is only a benign condition that does not require treatment [18]. By comparison, both of the diseases co-occurring in our patient were relatively serious. PCD is associated with cardiomyopathy and cardiac arrhythmia [19], while NICCD is correlated with liver disease [20]. The combination of inherited metabolic diseases may aggravate the clinical phenotype of the patient. Fortunately, early medical intervention leads to the long-term favourable prognosis of PCD. Consistent with previous studies [21], the main clinical presentation of the patient in this study was neonatal intrahepatic cholestasis. In addition, the patient had VSD, one of the most common congenital cardiac diseases in infants [22]. VSD is considered to have a relatively benign clinical course; however, sudden death can also occur in some cases [23]. To our knowledge, VSD has not been reported in patients with PCD, and this association remains unclear. Therefore, a long-term follow-up including the assessment of heart and liver function is necessary.

In summary, this study reported the first patient with both PCD and NICCD. The patient had extremely low C0 levels accompanied by a normal amino acid profile during NBS. The subsequent tests revealed neonatal intrahepatic cholestasis and VSD. Such dual disorders in a newborn broaden our understanding of inherited metabolic diseases. Thus, a long-term follow-up on the case is essential and is currently being performed.

## Supplementary information

**Additional file 1: Table S1.** The list of targeted genes

## Data Availability

The datasets used and/or analysed during the current study can be obtained from the corresponding author upon a reasonable request.

## Abbreviations

PCD: Primary carnitine deficiency
C0: Free carnitine
NBS: Newborn screening
NICCD: Neonatal intrahepatic cholestasis caused by citrin deficiency
MS/MS: Tandem mass spectrometry
NGS: Next-generation sequencing
VSD: Ventricular septal defect
