# Supplementary material for: Combined primary carnitine deficiency with neonatal intrahepatic cholestasis caused by citrin deficiency in a Chinese newborn
Source: BMC Pediatr. 2020 Oct 13;20:478. doi: 10.1186/s12887-020-02372-7 (PMC7552534; doi:10.1186/s12887-020-02372-7)
Supplement: Supplementary file 1 — Additional file 1: Table S1. The list of targeted genes [file 12887_2020_2372_MOESM1_ESM.docx]

Table S1. The list of targeted genes

| Number of genes | Targeted genes |
| --- | --- |
| 94 | *PAH, PTS, PCBD1, QDPR, SPR, GCH1, BCKDHA, BCKDHB, DBT, DLD, AMT, GCSH, GLDC, MAT1A, CBS, CTH, MTHFR, SUOX, MOCS1, MOCS2, GPHN, FAH, TAT, HPD, HGD, MUT, MMAA, MMAB, MMACHC, MMADHC, LMBRD1, ABCD4, MCEE, CD320, MLYCD, SUCLA2, SUCLG1, SUCLG2, PCCA, PCCB, GCDH, IVD, BTD, ACADSB, AUH, DNAJC19, CLPB, TMEM70, SERAC1, HMGCL, MCCC1, MCCC2, HLCS, ACADS, ACADM, ACADVL, HADH, HADHA, HADHB, ACAD8, ETHE1, ETFA, ETFB, ETFDH, ACAT1, SLC22A5, SLC25A20, CPT1A, CPT2, ARG1, ASL, ASS1, SLC25A13, CPS1, OAT, OTC, SLC25A15, SLC5A5, TPO, TG, TSHB, TSHR, PAX8, DUOX2, CYP11B1, CYP11B2, HSD3B2, STAR, CYP17A1, CYP11A1, POR, G6PD, ATP7B, PC* |
